# Facile Synthesis of Stable Cerium Dioxide Sols in Nonpolar Solvents

**DOI:** 10.3390/molecules27155028

**Published:** 2022-08-07

**Authors:** Alexander E. Baranchikov, Mikhail I. Razumov, Svetlana V. Kameneva, Madina M. Sozarukova, Tatiana S. Beshkareva, Arina D. Filippova, Daniil A. Kozlov, Olga S. Ivanova, Alexander B. Shcherbakov, Vladimir K. Ivanov

**Affiliations:** 1 Kurnakov Institute of General and Inorganic Chemistry of the Russian Academy of Sciences, 119991 Moscow, Russia; 2 Faculty of Chemistry, National Research University Higher School of Economics, 101000 Moscow, Russia; 3 Faculty of Materials Science, Lomonosov Moscow State University, 119991 Moscow, Russia; 4 Zabolotny Institute of Microbiology and Virology, National Academy of Sciences of Ukraine, 03680 Kyiv, Ukraine

**Keywords:** CeO_2_, antioxidants, hydrocarbons, fuel additives, chemiluminescence

## Abstract

A method is proposed for the preparation of stable sols of nanocrystalline cerium dioxide in nonpolar solvents, based on surface modification of CeO_2_ nanoparticles obtained by thermal hydrolysis of concentrated aqueous solutions of ammonium cerium(IV) nitrate with residues of 2-ethylhexanoic and octanoic acids. The synthesis was carried out at temperatures below 100 °C and did not require the use of expensive and toxic reagents. An assessment of the radical-scavenging properties of the obtained sols using the superoxide anion-radical neutralization model revealed that they demonstrate notable antioxidant activity. The results obtained indicate the potential of the nanoscale cerium dioxide sols in nonpolar solvents to be used for creating nanobiomaterials possessing antioxidant properties.

## 1. Introduction

Nanoscale cerium dioxide has a number of specific properties, including the ability to participate in reversible redox processes at physiological temperatures, extremely low solubility (p*K_sp_* ~ 59), the ability to mimic the functions of many natural enzymes, and low toxicity [1,2,3,4,5]. In this regard, it is the material of choice for a number of modern biomedical applications [6,7,8,9]. The biological activity of nanoscale CeO_2_ is governed by the high reactivity of its surface and the ability to interact with various organic compounds [10], as well as the high mobility of its oxygen sublattice [11,12,13]. It is notable that the question of the oxygen nonstoichiometry of nanocrystalline CeO_2_, which could explain its unique functional characteristics, is currently being actively discussed [14,15].

The broad range of possible biomedical applications of nanoscale CeO_2_ requires the development of synthetic approaches that ensure the production of cerium dioxide primarily as colloid solutions [1,15]. Because the therapeutic effect of CeO_2_ is achieved at sufficiently low concentrations (10^−5^–10^−7^ M) [16], the application of stable CeO_2_ sols ensures the required dosing accuracy.

Many methods have already been developed for the production of aqueous CeO_2_ sols, including sols stabilized by carboxylic acids, polysaccharides, etc. [17,18,19,20,21,22]. Normal and reverse precipitations, ion exchange, hydrothermal and hydrothermal–microwave treatment methods have been proposed to obtain such sols [17,18,19,20,21,22].

In contrast, almost no methods for the preparation of CeO_2_ sols in non-aqueous media (aprotic and nonpolar solvents) have been reported. In view of the high antioxidant and antibacterial activity demonstrated by nanoscale cerium dioxide, colloid solutions of CeO_2_ in aprotic organic solvents (primarily dimethyl sulfoxide) might be promising as preparations for the treatment of skin diseases, including skin cancer. It should be noted that the selective toxicity of nanodispersed CeO_2_ towards transformed cells has been reported in a number of reports [23]. Oil-dispersible ceria nanoparticles are also promising components of sunscreen compositions. The possibility of using cerium dioxide as a harmless alternative to traditional inorganic UV filters—titanium and zinc oxides—is currently being actively discussed [24,25,26]. Importantly, hydrophobic ceria nanoparticles are expected to be less prone to chemical interactions with organophosphates (e.g., phospholipids), which can block the redox cycling between Ce^+3^/Ce^+4^ in nanoceria [27], thus nullifying its positive antioxidant effect on the organic components of sunscreen formulations. Oil-dispersible ceria nanoparticles can also be used for the production of liposomal conjugates for advanced drug delivery systems and MRI contrast agents [28,29,30,31].

Taking into account the high catalytic activity of nanocrystalline CeO_2_ [32], non-aqueous colloid solutions of CeO_2_ are of particular interest for the production of diesel fuel additives. The purpose of such additives is to both reduce environmental pollution by inhibiting the emission of aerosols of ultrafine particulate matter in internal combustion engines (PM10) and increase fuel economy by reducing ignition delay and high flame sustenance [33,34,35,36,37,38]. A corresponding analysis has also been carried out for coarse-grained CeO_2_, but it is difficult to achieve a uniform distribution over the fuel volume for this material, due to the rapid sedimentation of ceria particles [36,38]. Based on cerium dioxide with a particle size of 7–15 nm, a commercially available catalyst for the afterburning of motor fuels, Envirox^TM^ (Oxonica, Aylesbury, UK), has been created [33,39,40,41], which has both high functional characteristics and rather low cytotoxicity and mutagenicity [42].

To the best of the authors’ knowledge, there have been reports of only two successful attempts to obtain stable sols of nanoscale cerium dioxide in nonpolar solvents based on the hydrothermal or solvothermal treatment of cerium(III) salts and fatty acids (2-ethylhexanoic and octanoic acids) [43,44]. Both methods are characterized by low scalability and require the use of high temperatures (up to 290 °C) and rather expensive starting compounds.

In this regard, the development of new facile methods for the preparation of cerium dioxide sols in nonpolar solvents is a relevant objective. To solve this problem, an approach is proposed that is based on modifying the surface of CeO_2_ nanoparticles obtained by the thermal hydrolysis of concentrated aqueous solutions of ammonium cerium(IV) nitrate [21] with residues of 2-ethylhexanoic and octanoic acids. The proposed synthetic sequence proceeds at temperatures below 100 °C and does not require the use of expensive and toxic reagents.

## 2. Experimental

### 2.1. Materials

The following reagents were used in the study: ammonium cerium(IV) nitrate (98.5%, Sigma-Aldrich (St. Louis, MO, USA) #22249), 2-ethylhexanoic acid (EHA, 99%, Sigma-Aldrich #E29141), octanoic acid (OA, 99%, Sigma-Aldrich #O3907), heptane (99%, Chimmed (Moscow, Russia)), ammonium bicarbonate (99%, Chimmed), isopropyl alcohol (IPA, 99.9%, Chimmed), xanthine (≥99.5%, Sigma-Aldrich #X0626), lucigenin (95%, J&K Scientific (San Jose, CA, USA), #393824), xanthine oxidase (≥0.4 units/mg protein, Sigma-Aldrich #X1875), potassium dihydrogen phosphate (Sigma-Aldrich, #P0662), potassium hydrogen phosphate (Sigma-Aldrich #P5655).

### 2.2. Synthesis of CeO_2_ Nanoparticles

For the synthesis of an aqueous cerium dioxide sol, a 0.18 M solution of (NH_4_)_2_[Ce(NO_3_)_6_] (2.30 g, 4.2 mmol) in distilled water (23 mL) was placed in a 100 mL Synthware™ autoclave and heated at 95 °C for 24 h. The resulting yellow precipitate was separated by centrifugation, washed three times with an excess of isopropyl alcohol and centrifuged again (20,000× *g*). The resulting paste was re-dispersed in 25 mL of distilled water and boiled for 2 h to remove the excess isopropanol. The concentration of CeO_2_ in the resulting sol was determined gravimetrically, after which it was diluted with distilled water to a concentration of 0.10 M CeO_2_. The parameters of the obtained aqueous CeO_2_ sols have been described by the authors in a previous report [21].

### 2.3. Surface Modification of CeO_2_ Nanoparticles

First, 0.75 g (0.84 mL, 5 mmol) of 2-ethylhexanoic acid or octanoic acid was mixed with 10 mL of heptane, followed by the addition of 0.4 g (5 mmol) of NH_4_HCO_3_ dissolved in 5 mL of distilled water under vigorous stirring. Then, 10 mL of aqueous ceria sol was added to the obtained mixture, followed by stirring at 40 °C in an open glass vessel until the complete evaporation of heptane. 10 mL of heptane was again added to the resulting sol, after which the mixture was vigorously shaken to extract CeO_2_ into the oil phase and the aqueous phase was removed on a separating funnel. As a result, a transparent yellow-green sol in heptane was obtained, which demonstrated a pronounced Tyndall effect. According to gravimetric estimates, the concentration of CeO_2_ in the resulting sols was 11–13 g/L.

### 2.4. Characterization Methods

Optical absorption spectra were recorded using quartz cuvettes (10.0 mm optical path length) in a 200–700 nm range at 0.2 nm steps on an OceanOptics (Orlando, FL, USA) QE-65000 spectrometer with deuterium-halogen (OceanOptics DH-2000) and xenon (OceanOptics HPX-2000) light sources.

X-ray powder diffraction patterns analysis (XRD) of sols previously dried at 40 °C was performed using a Bruker (Billerica, MA, USA) D8 Advance diffractometer (CuK_α_ radiation), in the angle range of 20–70°2θ, with a step of 0.03°2θ and a signal acquisition time of 0.7 s per step. Full-profile analysis of diffraction patterns was performed using TOPAS 4.2 software (Billerica, MA, USA) and diffraction maxima were approximated by Voigt pseudo-functions.

Dynamic light scattering (DLS) measurements were carried out using a Photocor (Tallinn, Estonia) Compact analyzer, which was equipped with a He-Ne laser (wavelength 632.8 nm) and enabled measurement of ζ-potential.

Transmission electron microscopy (TEM) and selected area electron diffraction (SAED) of the samples were conducted on a Carl Zeiss (Oberkochen, Germany) Leo912 AB Omega electron microscope operating at an accelerating voltage of 100 kV. Scanning transmission electron microscopy of the samples was performed using a Tescan (Brno, Česko) Amber GMH microscope at an accelerating voltage of 20 kV.

IR spectroscopic studies of the samples were performed in diffuse reflection geometry using an Infralum FT-08 spectrometer in the range of 400–4000 cm^−1^, with a resolution of 4 cm^−1^. The samples for the analysis were pressed into KBr pellets.

The radical-scavenging properties of the obtained materials were assessed using the chemiluminescent method with a model system generating superoxide anion radical (^•^O_2_^−^) using a DISoft (Moscow, Russia) Lum-100 single-channel chemiluminometer. The chemiluminescent signal was recorded in a phosphate buffer solution (100 mM, pH 7.4, K_2_HPO_4_) at 37 °C. Lucigenin was used as a chemiluminescent probe, xanthine oxidase was used as an initiator of free radical oxidation and xanthine was used as a chemiluminescent substrate. Background luminescence was recorded for 30–60 s after mixing solutions of xanthine (10 μM), lucigenin (50 μM) and the analyzed sample. Then, an aliquot of xanthine oxidase was added (0.11 units/mL, where 1 unit of activity corresponded to the conversion of 1.0 µmol of xanthine to uric acid for 1 min at 25 °C) and a chemiluminescent signal was recorded for 5 min. Light sums were calculated by numerical integration of chemiluminescent curves using PowerGraph software 3.3.11 (Moscow, Russia).

The analysis of the composition, structure and properties of the obtained materials was carried out using the equipment of the JRC PMR IGIC RAS.

## 3. Results and Discussion

During the hydrolysis of ammonium cerium(IV) nitrate at elevated temperatures, the formation of cerium dioxide nanoparticles takes place, which can form aqueous sols that are stable for at least several months after a decrease in the ionic strength of the medium [21]. The stability of such sols is due to the high value of the ζ-potential of the nanoparticle surface (+40 mV). Similar results indicating the high stability of aqueous CeO_2_ sols obtained by the hydrolysis of (NH_4_)_2_[Ce(NO_3_)_6_] in acidic media were reported by Pettinger et al. [45].

The high positive surface charge of CeO_2_ nanoparticles allows their modification with carboxylic acids, for example, fatty acids. Before carrying out the modification, 2-ethylhexanoic or octanoic acids were neutralized with ammonium bicarbonate.

**Figure 1:**
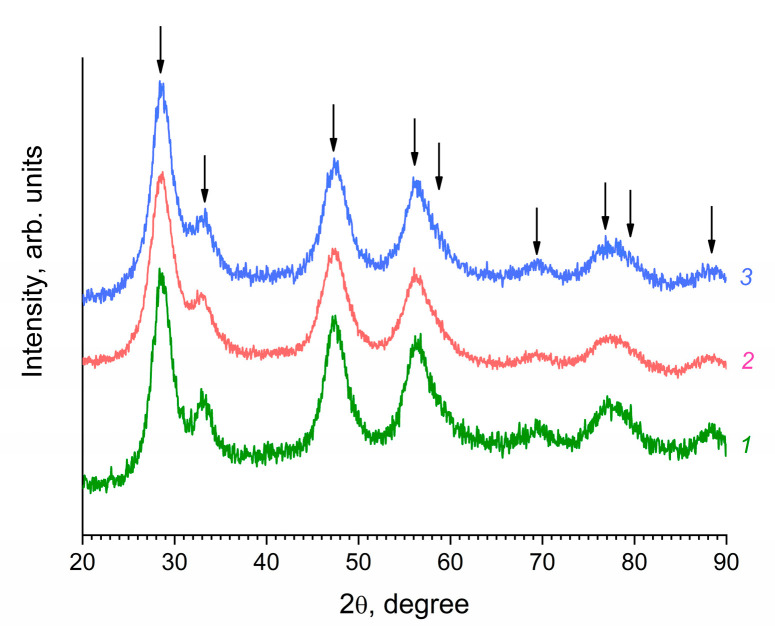
XRD patterns of the powders prepared upon the drying of (*1*) starting aqueous ceria sol and ceria sols in hexane stabilized by (*2*) 2-ethylhexanoic acid or (*3*) octanoic acid. Arrows indicate the positions of diffraction peaks corresponding to crystalline cerium dioxide (sp. gr. F$m\bar{3}m$, PDF2 #00-034-0394).

The XRD patterns of the unmodified and modified CeO_2_ sols dried at a low temperature (40 °C) are shown in Figure 1.

**Figure 2:**
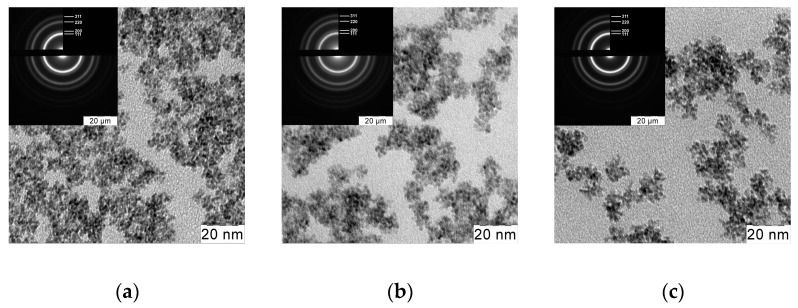
TEM images and SAED patterns of the (**a**) starting aqueous ceria sol and ceria sols in hexane stabilized by (**b**) 2-ethylhexanoic acid or (**c**) octanoic acid.

The data obtained indicated that all the obtained sols contained nanocrystalline cerium dioxide with a fluorite structure (sp. gr. $\mathrm{Fm}\bar{3}m$, PDF2 #00-034-0394). The crystallite size was established from the XRD data using the Scherrer formula, and was 3.0–3.5 nm. Transmission electron microscopy data confirmed the results of XRD, indicating the formation of nanoparticles with a high degree of crystallinity and a relatively low degree of aggregation during the synthesis (Figure 2).

**Figure 3:**
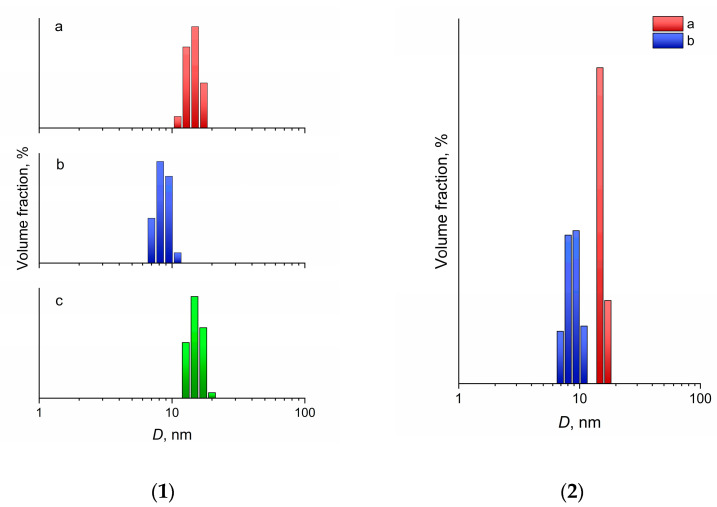
(**1**) Hydrodynamic diameter distributions, as measured using DLS, for ceria nanoparticles in hexane stabilized by (**a**) 2-ethylhexanoic acid, (**b**) octanoic acid, and (**c**) the initial aqueous ceria sol as a reference. (**2**) Dynamic light scattering results for the same sols after drying, followed by re-dispersion in heptane.

The DLS analysis of the sols (Figure 3) also confirmed the XRD and TEM data. CeO_2_ sols in heptane stabilized with 2-ethylhexanoic and octanoic acids contained weakly aggregated nanoparticles with an average hydrodynamic diameter of 15.6 and 9.2 nm, respectively. The average hydrodynamic diameter of particles in the initial aqueous CeO_2_ sol was 16 nm. The marginally larger particle size recorded using the DLS method, compared with the size determined using XRD and TEM, was obviously associated with the presence of an electrical double layer, as well as the presence of adsorbed molecules and ions on the surface of CeO_2_ nanoparticles.

It is notable that the size of the 2-ethylhexanoate anion was ~0.8–0.9 nm [46,47] and the size of the octanoate anion was 1.1–1.2 nm [48,49,50], but the hydrodynamic diameter of CeO_2_ nanoparticles obtained using 2-ethylhexanoic acid was approximately 1.5 times larger than the diameter of nanoparticles obtained using octanoic acid. This size difference is most likely the result of linear octanoate anions forming a denser layer on the surface of nanoparticles, thus preventing their aggregation more effectively. Less aggregation of CeO_2_ nanoparticles stabilized with octanoic acid, compared with those stabilized with 2-ethylhexanoic acid, was indirectly confirmed by the range of the hydrodynamic diameters’ distributions at half maximum; these were 2.2 and 4.0 nm, respectively.

**Figure 4:**
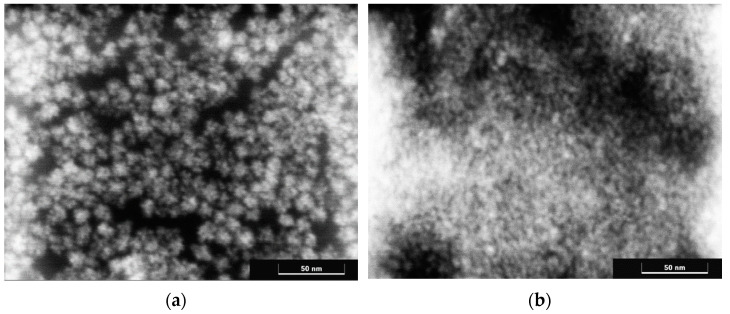
Micrographs of cerium dioxide sols stabilized with (**a**) 2-ethylhexanoic acid and (**b**) octanoic acids obtained by scanning transmission electron microscopy.

Scanning transmission electron microscopy data confirmed slightly more nanoparticle aggregation in sols stabilized with 2-ethylhexanoic acid (Figure 4). For such sols, aggregates about 10 nm in size were observed to consist of smaller nanoparticles.

The ability of nanoparticles to be re-dispersed after complete removal of the liquid is an important characteristic of colloid solutions, which is of great importance for their practical application. Dry nanoparticles are much more convenient in transportation and storage, and less stringent packaging requirements are imposed on them. In particular, the transportation of organic solvents is associated with significant risks due to their high flammability and low flash points. Thus, n-heptane is a highly flammable liquid, with a flash point of −4 °C and an auto-ignition temperature of 204 °C. As a result, cerium dioxide sols in heptane were dried at a temperature of 40 °C to a constant weight, after which heptane was added to them in a volume that was equal to the initial volume. CeO_2_ nanoparticles stabilized with both 2-ethylhexanoic and octanoic acids were completely transferred into the liquid phase, forming transparent sols, as confirmed by the results of DLS analysis (Figure 3). The average hydrodynamic diameter of the nanoparticles actually did not change after the re-dispersion of the sols, being 15.4 nm for the sols stabilized with 2-ethylhexanoic acid and 9.5 nm for the sols stabilized with octanoic acid.

Thus, the proposed synthetic method also enables the production of cerium dioxide powders modified with fatty acids that can be completely re-dispersed in nonpolar solvents.

**Figure 5:**
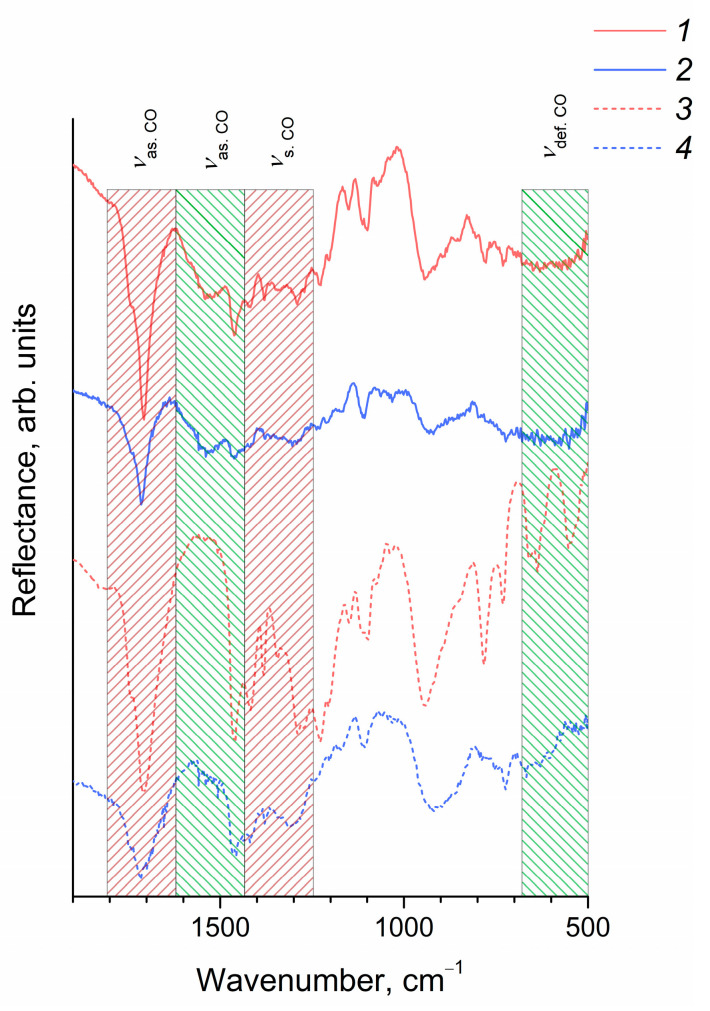
The IR spectra of CeO_2_ sols in heptane stabilized with (*1*) 2-ethylhexanoic and (*2*) octanoic acids. The IR spectra of (*3*) 2-ethylhexanoic and (*4*) octanoic acids are shown for comparison.

The IR spectroscopy data (Figure 5) confirmed the chemical immobilization of octanoic and 2-ethylhexanoic acid residues on the surface of CeO_2_ nanoparticles. In the IR spectra of CeO_2_ nanoparticles, as well as octanoic and 2-ethylhexanoic acids, intense bands were present at 1716 cm^−1^ and 1708 cm^−1^, respectively, corresponding to antisymmetric stretching vibrations of the carboxyl group; bands were also observed in the range of 1420–1460 cm^−1^, corresponding to symmetric stretching vibrations of the carboxyl group. At the same time, the IR spectra of CeO_2_ nanoparticles included a broad band at ~1540 cm^−1^, which was absent in the spectra of carboxylic acids and can be attributed to antisymmetric stretching vibrations of the carboxyl group coordinated with a metal cation [51,52]. A comparison of the mutual arrangement of bands in the IR spectra of CeO_2_ nanoparticles related to symmetric and antisymmetric vibrations of the carboxyl group indicated the chelate nature of the coordination of COO-groups with respect to the cerium cation where both oxygen atoms coordinate a single metal ion [53]. The intensity of the bands in the range of bending vibrations of the carboxyl group (530–550 and 630–670 cm^−1^), for both modified CeO_2_ sols, was significantly lower than for pure acids, which was probably due to the hindered rotation of the carboxyl group coordinated with the bulk CeO_2_ nanoparticle.

**Figure 6:**
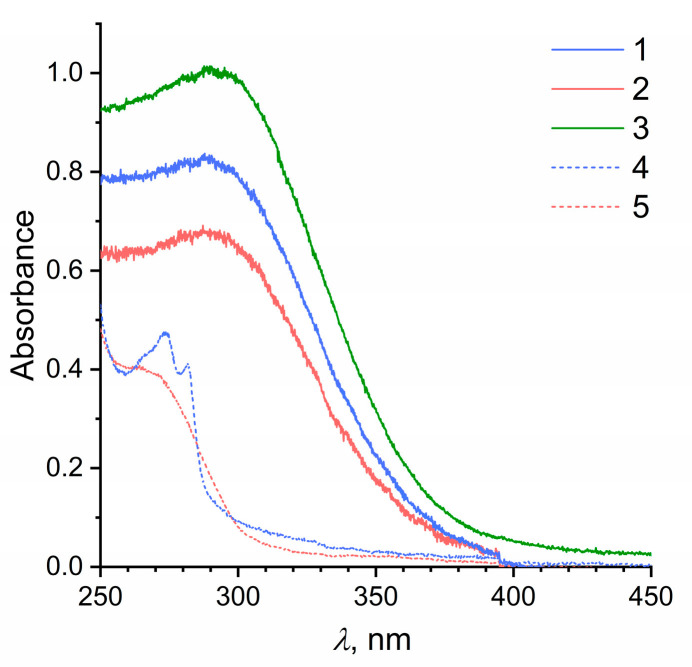
Optical absorption spectra of CeO_2_ sols in heptane stabilized with (*1*) octanoic acid or (*2*) 2-ethylhexanoic acid; (*3*) CeO_2_ aqueous sol. The optical absorption spectra of (*4*) octanoic acid and (*5*) 2-ethylhexanoic acid solutions are shown for comparison.

The optical absorption spectra of nanocrystalline CeO_2_ sols stabilized with 2-ethylhexanoic or octanoic acid in heptane (Figure 6) are typical of cerium dioxide sols; they demonstrate their ability to absorb radiation with a wavelength of less than ~400 nm, which corresponds to the 3.0 eV band gap of cerium dioxide [54]. The optical absorption spectra of CeO_2_ sols in heptane did not show a noticeable increase in optical density in the range of 250–300 nm compared with the aqueous CeO_2_ sol, which indirectly confirmed the absence of significant amounts of free 2-ethylhexanoate or octanoate anions.

The ability of cerium dioxide to absorb UV radiation makes this material highly promising for application as UV filters in sunscreens [24,25,26,55,56,57]. An additional advantage of cerium dioxide is its significant ability to inactivate reactive oxygen species, including hydrogen peroxide, radical anion superoxide, singlet oxygen, and hydroxyl radicals [58,59,60]. Aqueous sols of nanocrystalline cerium dioxide stabilized with organic acids, polysaccharides, etc., also demonstrate high antioxidant activity [58,59,60]. At the same time, equivalent studies of CeO_2_ nanoparticles stabilized by amphiphilic ligands are currently absent, although they are of key importance for the design of new biologically active oil-soluble preparations that protect the skin from various damaging factors and that can be used for the treatment of skin diseases.

To analyze the antioxidant properties of cerium dioxide sols in heptane, a model system based on xanthine, xanthine oxidase, and lucigenin was chosen that ensured the generation of superoxide anion radicals in an aqueous medium. The selection of the volumetric ratios of the components made it possible to create fairly stable thin emulsions, which, in turn, provided a reproducible analytical signal during the analysis of the samples.

**Figure 7:**
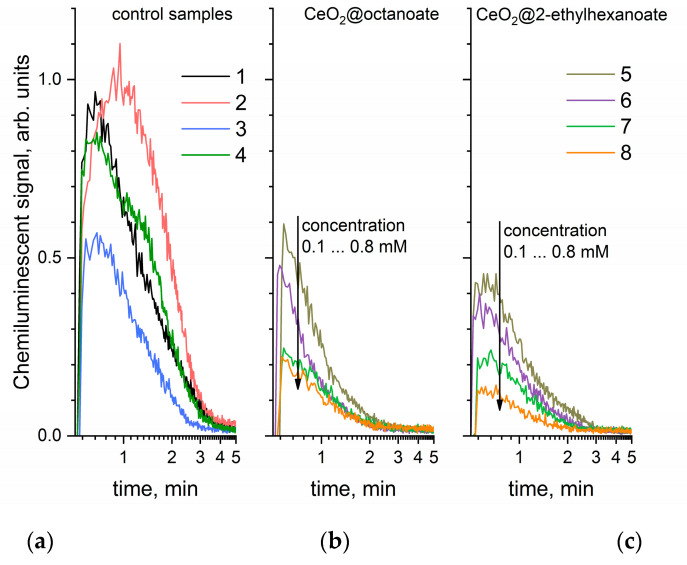
Chemiluminograms obtained by introducing xanthine oxidase into a system containing xanthine, lucigenin, and (**a**) control samples, and CeO_2_ sols stabilized with (**b**) octanoic and (**c**) 2-ethylhexanoic acids. The reference samples used: (1) a phosphate buffer solution; (2) a solution of octanoic acid in heptane (4.0 mM); (3) a solution of 2-ethylhexanoic acid in heptane (4.0 mM); (4) heptane. CeO_2_ sols in heptane with concentrations (5) 0.1 mM, (6) 0.2 mM, (7) 0.4 mM and (8) 0.8 mM were used for the experiments. In all the panels, the same y-axes ranges are used.

Figure 7 presents chemiluminograms, whose integration indicates the amount of superoxide anion radical formed in the test system after adding xanthine oxidase.

The data presented in Figure 7 indicate that cerium dioxide sols significantly suppress chemiluminescence resulting from the generation of the superoxide anion radical which is formed during the oxidation of xanthine catalyzed by xanthine oxidase in the presence of oxygen. It is notable that octanoic acid by itself also slightly reduced the concentration of the superoxide anion radical, while the addition of 2-ethylhexanoic acid led to a small increase in total chemiluminescence intensity. The absence of pronounced antioxidant properties in saturated fatty acids with a hydrocarbon chain length <10 corresponds to previously published data [61]. The experimental results obtained suggest that the observed antioxidant effect was caused precisely by the presence of nanoscale cerium dioxide in the sols investigated.

A calculation of the relative degree of chemiluminescence suppression as a function of sol concentration was performed to compare the antioxidant activity of CeO_2_ colloid solutions stabilized with 2-ethylhexanoic and octanoic acids ($\frac{C_{0}-C}{C}\cdot 100\%$, where *C*_0_ and *C* are the total chemiluminescence intensities for the control and analyzed samples, respectively).

**Figure 8:**
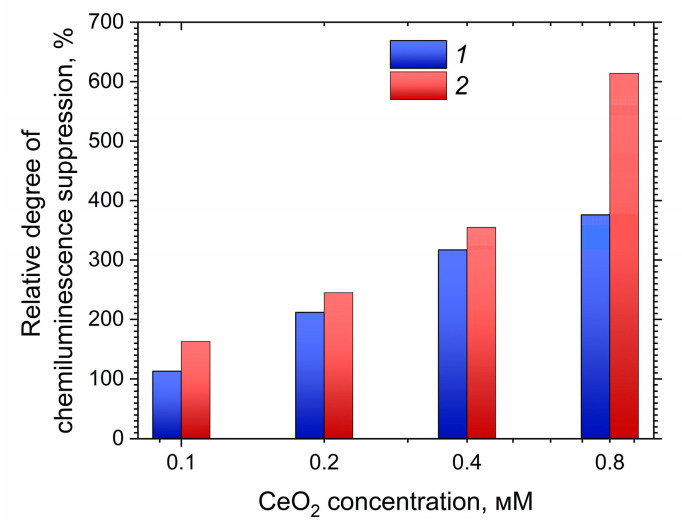
Comparison of the antioxidant activity of nanocrystalline CeO_2_ sols stabilized with (1) octanoic and (2) 2-ethylhexanoic acids with respect to the superoxide anion radical.

The data presented in Figure 8 indicate that CeO_2_ sols stabilized with 2-ethylhexanoic acid demonstrated greater antioxidant activity than CeO_2_ sols stabilized with octanoic acid over the entire range of concentrations studied. This result also supports the conclusion that the antioxidant activity of the sols was determined precisely by the cerium dioxide nanoparticles, and not by their ligand environment. It is likely that the different activities of the sols are associated with different orientations of the ligands relative to the surface of the nanoparticles. A less dense layer of 2-ethylhexanoic acid residues encourages a higher level of chemical activity on the surface of nanoparticles, despite their higher degree of aggregation.

## 4. Conclusions

A simple low-temperature method for the preparation of re-dispersible sols of nanocrystalline cerium dioxide in nonpolar solvents (using heptane as an example) has been proposed. 2-ethylhexanoic and octanoic acids have been used as stabilizers for colloid CeO_2_ particles. The size of CeO_2_ crystallites in the obtained sols was ~3 nm, with an average hydrodynamic diameter of ~10–15 nm. The radical-scavenging properties of the materials obtained, with respect to the superoxide anion radical, have been evaluated using the xanthine–lucigenin–xanthine oxidase test system. It has been shown that CeO_2_ nanoparticles stabilized with 2-ethylhexanoic and octanoic acids significantly suppress chemiluminescence in this system, which indicates their pronounced antioxidant activity. The results obtained suggest the potential of the obtained nanomaterials for application in advanced UV filters that have a pronounced antioxidant effect. Oil-dispersible ceria nanoparticles can be considered as an alternative to traditional inorganic UV filters (titanium and zinc oxides). They can also be considered for the production of liposomal conjugates for advanced drug delivery systems and MRI contrast agents.

## Data Availability

Data is contained within the article.

## Sample Availability

Samples of the CeO_2_ sols in heptane are available from the authors.
